# Age Differences in the Association of Severe Psychological Distress and Behavioral Factors with Heart Disease

**DOI:** 10.1155/2013/979623

**Published:** 2013-03-17

**Authors:** Liang Wang, Ke-Sheng Wang

**Affiliations:** Department of Biostatistics and Epidemiology, College of Public Health, East Tennessee State University, P.O. Box 70259, Lamb Hall, Johnson City, TN 37614-1700, USA

## Abstract

Few studies have examined the risk factors of serious psychological distress (SPD) and behavioral factors for heart disease separately stratified as young (18–44 years), middle aged (45–64 years), and elderly (65 years or older). A total of 3,540 adults with heart disease and 37,703 controls were selected from the 2005 California Health Interview Survey. Data were weighted to be representative and adjusted for potential undercoverage and nonresponse biases. Multiple logistic regression models were used to estimate the associations of the factors with heart disease at different ages. The prevalence of SPD was 8% in cases and 4% in controls, respectively. For young adults, SPD and higher federal poverty level (FPL) were associated with an increased risk of heart disease while for middle-aged adults, SPD, past smoking, lack of physical activity, obesity, male, and unemployment were associated with an increased risk of heart disease. In addition, SPD, past smoking, lack of physical activity, obesity, male, unemployment, White, and lower FPL were associated with an increased risk of heart disease in elderly. Our findings indicate that risk factors for heart disease vary across all ages. Intervention strategies that target risk reduction of heart disease may be tailored accordingly.

## 1. Introduction

Cardiovascular disease (CVD) is the leading cause of death for adults in the world [1, 2]. Although the death rate for CVD has declined by 30.6% from 1998 to 2008, coronary heart disease (CHD) as the main component of CVD remains the leading cause of death in the United States (USA) for men and women and particularly is the single leading killer in women [3, 4]. In 2010, the age-adjusted prevalence of heart disease for adults was 11.7% among Whites, 10.9% among Blacks or African Americans, 8.1% among Hispanics or Latinos, and 7.2% among American Asians [5]. Approximately half men and one-third women aged 40 years or older were at a higher risk of developing CHD [6]. CHD accounts for more than 50% of all cardiovascular events for both sexes younger than 75 years of age [7]. The estimated direct and indirect costs of heart disease in 2008 were $190.3 billion [4]. Furthermore, approximately four of every ten individuals in the USA are predicted to have some form of CVD by 2030 [8].

Several risk factors have been linked to predispose individuals to develop heart disease, including gender, heredity, age, physical inactivity, smoking, obese status, high blood pressure, high cholesterol, abnormal blood lipid levels, low daily fruit and vegetable consumption, alcohol drink, psychosocial index, and diabetes [9, 10], where the combined psychosocial index was based on scores to five interrelated items including depression status, general stress (home or work), financial stress, number of life events, and locus of control [10]. Studies on the association between mental health problems and heart disease have been conducted. For example, depression and depressive symptoms were associated with CHD [11, 12], ventricular arrhythmia, and with sudden cardiac death [13–17]. Worry was highlighted in a growing literature with the development of CHD [18]. The association between Type D personality and progression of CHD is well established [19]. Psychological distress has been reported in England to be associated with an increased risk of CHD [20]. However, few studies have focused on serious psychological distress (SPD) with heart disease [21]. Among the risk factors that cannot be changed (e.g., gender, race, and age), many studies have only evaluated gender or race differences in risk factors for heart disease. For example, risk factors were examined by relative risk and population-attributable risk in men and women [22]. Differences in risk factors for CHD were found among ethnic groups such as foreign born Afro-Caribbeans, USA born Afro-Caribbean Americans, and American Americans [23]. The prevalence of having two or more risk factors of six risk factors (high blood pressure, high cholesterol, diabetes, current smoking, physical inactivity, and obesity) was the highest among Blacks (48.7%) and American Indians/Alaska Natives (46.7%) and the lowest among Asians (25.9%) [24]. However, age-based variations should be noticed for the risk factors. For example, adults aged 18–44 years (59.9%) were more likely to have regular physical activity than those aged 45–64 years (55.3%) and those aged 65 years or older (48.5%); the percentage of healthy weight is higher in young adults aged 18–44 years (43.7%) when compared with that in middle-aged adults aged 45–64 years (31.4%) and in elderly aged 65 years or older (37.3%); and the prevalence of current nonsmokers was higher in elderly aged 65 years or older (89.7%) than that in middle-aged adults aged 18–44 years (76.1%) and in young adults aged 45–64 years (77.7%) [4]. These variations may contribute to the hypothesis that the predictors of heart disease may be different in each age stratum. To our knowledge, limited studies have examined age differences in risk factors of SPD and behavioral factors (such as smoking and physical activity) for heart disease particularly in USA adults. The specific aim of this study was to examine the risk factors for heart disease at three ages: young (18–44 years), middle aged (45–64 years), and elderly (65 years or older). If confirmed, prevention programs that target risk reduction are particularly critical to the burden of heart disease in the high-risk USA adults at each age. 

## 2. Materials and Methods

### 2.1. Data Source

The California Health Interview Survey (CHIS) is a population-based geographically stratified telephone survey of California's population conducted every other year since 2001. The CHIS is the largest health survey conducted in any state and one of the largest health surveys in the country. The cross-sectional survey consisted of two stages: (1) a sample of telephone numbers was selected by use of a list-assisted random-digit-dial method, (2) one adult who was 18 years of age or older was randomly selected among all adults in the household as the respondent. Serious psychological distress was assessed by Kessler 6 (K6) scale, which has been widely used to screen for Diagnostic and Statistical Manual of Mental Disorders Fourth Edition (DSM-IV) mood and anxiety disorders in the general population [25, 26], and may show a higher specificity (0.96) than previous studies on assessment of psychological distress [27]. The methods were described in detail elsewhere [28, 29]. CHIS 2005 is the third CHIS data collection cycle and was conducted between July 2005 and April 2006, where CHIS completed interviews with 43,020 adults statewide with extensive information for all age groups on health status (general health status, height and weight, and days missed from school due to health problems), health conditions (asthma, diabetes, heart disease, high blood pressure, epilepsy, and physical disability/need for special equipment), health-related behaviors (dietary intake, physical activity and exercise, walking for transportation and leisure, flu shot, alcohol and tobacco use, sexual behavior, STD testing, and birth control practices), health insurance coverage, access to health care services, and other health and health-related issues [30]. The sample was weighted to represent the noninstitutionalized population for each sampling stratum and statewide. Complete information in detail on the weighting procedures can be found at http://healthpolicy.ucla.edu/chis/design/Documents/CHIS2005_method5.pdf. The response rate for the adult extended interview was 54%. Complete information in detail on CHIS response rates can be found at http://healthpolicy.ucla.edu/chis/design/Documents/CHIS2005_method4.pdf. In this study, subjects who were Pacific Islander (*n* = 120; 0.28%), American Indian/Alaskan native (*n* = 554; 1.29%), and other single/multiple race (*n* = 1103; 2.56%) were excluded due to the small sample size. A total of 41,243 participants were included for the final analysis.

Procedures for data collection and analysis were approved by the Institutional Review Boards (IRBs) at the University of California, Los Angeles, and the California Health and Human Services Agency. All the participants provided written and informed consent for their participation. The current study was approved by the IRB of East Tennessee State University. 

### 2.2. Dependent Variable

Heart disease outcome was determined by the question “Have doctors ever told adults that they have any kind of heart disease?” and dichotomized into either yes or no.

### 2.3. SPD and Behavioral Factors

SPD was assessed using the K6 scale [27], which comprises 6 questions asking how often during the past 30 days a person felt “so sad that nothing could cheer them up,” “nervous,” “restless,” “hopeless,” “worthless,” or that “everything was an effort.” Responses were scored from 0 (none of time) to 4 (all the time). Compared to other instruments (e.g., the World Health Organization's Disability Assessment Schedule), the K6 showed a high total classification accuracy (0.96) and was the most efficient screening tool [27]. A total score was summed and ranged from 0 to 24. SPD was defined as a score of ≥13 [31]. Smoking status was divided into three categories: never smoking, current smoking, and past smoking. Other behavior factors were dichotomized into either yes or no, including binge drink, physical activity, and obesity. For binge drink, males and females were assigned to yes if they had five drinks or more and four drinks or more, respectively. Physical activity was determined by the question “Whether engage in moderate or vigorous physical activity in past week?” Body Mass Index (BMI) was calculated as weight in kilograms divided by squared height in meters. Obesity for adults was defined as BMI was 30.0 or above [32].

### 2.4. Demographic and Other Factors

Gender was self-reported as either male or female. The age was categorized into young (18–44 years), middle aged (45–64 years), and elderly (65 years or older), as consistent with other studies [4]. Employment status was dichotomized into either yes or no. Originally, there were six racial groups in the study including White, Latino, Asian, African American, Pacific Islander, and American Indian/Alaskan native. Because of the small sample size for Pacific Islander, American Indian/Alaskan native, and other single/multiple race, only White, Latino, Asian, and African American subjects were included. The Federal Poverty Level (FPL) is an indicator comprised of a single national set of levels for families of various sizes. Poverty status for a family is calculated by comparing FPL to unadjusted gross income. The poverty amounts were calculated as the product of the cost of minimal food budget multiplied by 3 and were calibrated using the Consumer Price Index (CPI), which does not change the FPL nor does it raise standards of living [33]. Poverty level was divided into four categories: 300% FPL or above, 0–99% FPL, 100–199% FPL, and 200–299% FPL. 

### 2.5. Statistical Analysis

In this study, all the variables were categorical. Thus, the sample characteristics were described using counts with percentages. The proportions of subjects (cases and controls) for all potential risk factors (SPD, smoking status, binge drink, physical activity, obesity, gender, age, employment, race, and FPL) were weighted by using the SAS PROC SURVEYFREQ procedure [34], which provides weighted cross-tabulation tables. By using the SAS PROC SURVEYLOGISTIC procedure [34], we fit logistic regression to estimate odds ratios (ORs) and 95% confidence intervals (CIs) for the relation between potential risk factors and binary heart disease outcome. We fit two models for analysis. In model one, simple logistic regressions were used to examine the independent roles of potential risk factors in heart disease; multiple logistic regressions then were used to simultaneously adjust for all found significant factors of heart disease in the univariate analysis. In model two, to determine different risk factors at each age, we fit multiple logistic regressions to adjust for SPD, smoking status, binge drink, physical activity, obesity, gender, employment, race, and FPL stratified as the three ages (28–44 years, 45–64 years, and 65 years or older). All the analyses were performed with SAS statistical software, version 9.2 (SAS Institute, Cary, NC, USA) [34]. *P* values <0.05 on two-side tests were considered statistically significant. 

## 3. Results

### 3.1. Subjects Characteristics

The prevalence of SPD was 8% in cases and 4% in controls, respectively. In both cases and controls, almost half subjects were males, and the majority of subjects did not binge drink, had lack of physical activity, were absent of SPD, were obese, were White or Latino, and lived at higher FPL (Table 1). In cases, the prevalence of never smokers and past smokers were 45% and 43%, respectively. More people were 65 years or older (53%) and unemployed (73%). In controls, the prevalences of never smokers and past smokers were 62% and 23%, respectively. Less people were 65 years or older (12%) and unemployed (38%).

### 3.2. Relationships between All Potential Risk Factors and Heart Disease

Simple logistic regression showed that all the potential risk factors were significantly associated with heart disease (all *P* < 0.05) (Table 2). After adjusting for all other factors, SPD was associated with about twice more likelihood of having heart disease (OR = 2.15, 95% CI = 1.70–2.72). Compared to subjects who were never smokers, subjects who were past smokers were more likely to have heart disease (OR = 1.41, 95% CI = 1.25–1.57). Subjects who engaged in physical activity were less likely to develop heart disease (OR = 0.77, 95% CI = 0.67–0.87). Obese adults were more likely to have heart disease than nonobese adults (OR = 1.34, 95% CI = 1.19–1.52). Females were less likely to have heart disease (OR = 0.70, 95% CI = 0.63–0.78). Compared to subjects aged 18–44 years, subjects aged 45–64 years were four times more likely to develop heart disease (OR = 4.0, 95% CI = 3.32–4.83), and subjects aged 65 years or older were almost eleven times more likely to develop heart disease (OR = 10.98, 95% CI = 9.18–13.15). Employment was associated with a reduced risk of heart disease (OR = 0.51, 95% CI = 0.45–0.59). Compared to Whites, Latinos were less likely to develop heart disease (OR = 0.70, 95% CI = 0.57–0.86). Subjects living at 0–99% FPL were more likely to develop heart disease than those living at 300% or above FPL (OR = 1.25, 95% CI = 1.04–1.50) (Table 2). 

### 3.3. Age Differences in Risk Factors for Heart Disease

Age differences in risk factors for heart disease are presented in Table 3. Generally, SPD was associated with an increased risk of heart disease especially in young age and middle-aged group, whereas smoking, physical activity, obesity, gender, and employment were associated with heart disease in the middle-aged and elder groups. Specially, for subjects aged 18–44 years, SPD was associated with an increased risk of heart disease (OR = 2.29, 95% CI = 1.26–4.17). Compared with subjects living at 300% FPL or above, subjects living at 200–299% FPL were less likely to develop heart disease (OR = 0.49, 95% CI = 0.25–0.96). For subjects aged 45–64 years, compared to never smokers, past smokers were more likely to have heart disease (OR = 1.44, 95% CI = 1.19–1.76). SPD and obesity were associated with an increased risk of heart disease (OR = 2.24, 95% CI = 1.62–3.09; OR = 1.45, 95% CI = 1.17–1.79, resp.). Physical activity, female, and employment were associated with a reduced risk of heart disease (OR = 0.79, 95% CI = 0.64–0.98; OR = 0.69, 95% CI = 0.57–0.85; OR = 0.47, 95% CI = 0.39–0.57). For subjects aged 65 years or older, compared with never smokers, past smokers were more likely to have heart disease (OR = 1.37, 95% CI = 1.18–1.59). SPD and obesity were associated with an increased risk of heart disease (OR = 1.81, 95% CI = 1.14–2.88; OR = 1.27, 95% CI = 1.06–1.52, resp.). Physical activity, female, and employment were associated with a reduced risk of heart disease (OR = 0.67, 95% CI = 0.57–0.80; OR = 0.60, 95% CI = 0.52–0.70; OR = 0.48, 95% CI = 0.36–0.63). Compared to White, Latino and African American were less likely to have heart disease (OR = 0.54, 95% CI = 0.36–0.79; OR = 0.68, 95% CI = 0.49–0.94, resp.). Compared to subjects living at 300% FPL or above, subjects living at 0–99% FPL were more likely to develop heart disease (OR = 1.58, 95% CI = 1.20–2.07).

## 4. Discussion

In this study, we demonstrated the age differences in risk factors for heart disease. In summary, SPD and lower FPL were associated with an increased risk of heart disease for young adults. SPD, past smoking, lack of physical activity, obesity, male, and unemployment were associated with an increased risk of heart disease for middle-aged adults. SPD, past smoking, lack of physical activity, obesity, male, unemployment, White, and lower FPL were associated with an increased risk of heart disease for elderly. 

For behavioral factors, past smoking was associated with a higher risk of heart disease in middle aged and elderly but not in young. Studies have shown a relationship between cigarette smoking and heart disease [24, 35] and proposed a potential mechanism that smoke exposure increases oxidative stress for interrupting cardiovascular function [36]. However, oxidative stress is more common in older subjects [37]. Physical activity was found to be a protective predictor of heart disease at older ages, as consistent with previous studies that heart disease occurred at a later age and tended to be less severe in individuals with physical activity [38–40]. Consistent with previous evidence [41], our study showed that obese adults were more likely to have heart disease, but only for those at older ages, which may indicate a long-term impact of obesity on the risk of heart disease.

Environmental stress factors and “internal vulnerability” factors have been suggested as risk factors for heart disease. For instance, some physical (e.g., physical activity, sexual activity), psychological (e.g., anger, depression, anxiety, frustration, work stress, earthquakes, war, and terror attacks), chemical (e.g., coffee, alcohol consumption), and environmental (e.g., pollution) triggers have been examined [42]. However, the evidence for the relation of SPD to heart disease has been rare. In the current study, SPD was found to have a significant increased likelihood of heart disease at all three ages. Psychological distress has been reported in England to be associated with an increased risk of CHD [20] and of mortality [43]. Even a general propensity to psychological distress can be linked to adverse cardiovascular events [44]. Although SPD was shown to be associated with CVD among Colorado adults [21], to our knowledge, this is the first study to evaluate the roles of SPD in developing heart disease in USA adults at different ages. In addition, our findings of consistent increased likelihood of heart disease due to SPD in the lifetime and of a higher risk in young and middle aged than elderly may imply a strategy of reducing heart disease that psychological distress needs to be prevented at all ages particularly at younger ages.

For demographic and other factors, males aged 45 years or older were at a higher risk of developing heart disease. Males may be subject to unhealthy behaviors such as smoking, alcohol, and meat consumption, but they were still at a higher risk of heart disease after adjusting for these behaviors [45], as consistent with our finding. Employment was associated with a reduced risk of heart disease in middle aged or in elderly. Risk of heart disease was showed to be related to employment in men, but not consistent in women [46, 47]. Employed subjects may have healthier lifestyles and behavioral factors such as more exercise or less smoking due to higher education, income level, and awareness of health. Compared with elderly Whites, elderly Latinos and African Americans were at a lower risk of heart disease, which is inconsistent with prior studies which found that Blacks were at a higher risk of heart disease in their 30s–50s [48]. This explanation may be that, compared to elderly, young or middle aged may be less likely to be insured, access to medical care and afford medications [49–52] or be reluctant to adhere to medications. These difficulties may become more obvious by lower awareness among young and middle-aged adults of their heart disease risk and reluctance on the treatment of CVD risk factors at younger ages [53, 54]. Lower poverty level was found to be associated with a decreased risk of heart disease in young age, but an increased risk of heart disease in elderly. This finding may be in parallel with inconsistent suggestions from prior studies. For example, one study suggested that poor status was detrimental to heart disease due to the adverse effects of behaviors such as smoking [55], while another study claimed that low socioeconomic deprivation was shown to protect against smoking [56].

The findings in the present study suggest that prevention strategies should be tailored to people at each age because differences in the various risk factors were observed among the three age groups. The findings were different from a study in Denmark, which examined relative risks (RRs) and population-attributable risks (PARs) stratified on ages and found that most risk factors had a similar risk of CHD in the young (30–54 years), middle ages (55–64 years), and elderly (65–79 years) [22]. In the current study, however, only SPD was found to be associated with heart disease across all ages. The different findings between our study and the Denish study may be due to different population characteristics, stratified ages, risk factors of interest, and measures of risk.

### 4.1. Strengths and Limitations

This study presents strengths. First, using a population-based random-digit-dial telephone survey, a large sample size of subjects from 2005 CHIS were widely selected by comprehensive information for both sexes and the wide age range on heart disease and other health-related issues. Second, data were collected by well-trained staff using five languages to capture the rich diversity of the population, and weighted to be representative of the population. Two different imputation procedures were used to address missing responses for items important for weighting the data. 

However, there are several limitations in the current study. First, subjects who did not have phones or did not respond to the calling were excluded from the survey. Currently, many people particularly younger people have no longer landlines at all as they use cell phones exclusively, which may cause a selection bias. In addition, institutionalized people who were homeless or lived in group homes, nursing home, or prisons were excluded. The exclusions may be more likely to limit the number of elderly and underestimate the risk factors of heart disease in this age. Second, self-report rather than objective measures may be more likely to result in recall bias, which may have underestimated the prevalence of heart disease. Despite a possible exposure misclassification, we believe that evidence supports that this would be a nondifferential misclassification. However, such misclassification can cause loss of statistical power and may partly explain the differences in results. Third, given the evidence of poor concordance between self-reports and medical records [57, 58], the current study did not check the veracity of health data. Fourth, the response rate for the interview was 54%. However, CHIS data are high quality and accurately represent California's household population. In this study, CHIS nonresponse bias is assessed using administrative data to compare neighborhood characteristics among respondents and nonrespondents. CHIS 2005 administrative data that included both respondents and nonrespondents was linked to 2000 USA Census data at the census tract level. Results show little to no substantial differences in neighborhood characteristics between respondents and nonrespondents [59]. Finally, this is the cross-sectional survey and represents a “snapshot” of risk factors and heart disease at one time point in time and limits the ability to determine cause and effect. For instance, participants were likely to have followed their doctors' advice and started being more active after being diagnosed with heart disease, which may partly explain the relationship between current smoking behavior and heart condition.

## 5. Conclusions

Overall, SPD, past smoking, lack of physical activity, obesity, male, older ages, unemployment, White, and lower FPL were associated with an increased risk of heart disease. However, risk factors for heart disease varied as young, middle aged, and elderly. Intervention strategies that target risk reduction of heart disease may be tailored accordingly. 

## Figures and Tables

**Table 1:** Subjects characteristics using 2005 California Health Interview Survey (*N* = 41,243).

Variables	Cases	Controls
	*n* (weighted %)	*n* (weighted %)
SPD and behavioral factors		
SPD		
No	3263 (92%)	36305 (96%)
Yes	230 (8%)	1308 (4%)
Smoking status		
Never	1521 (45%)	21663 (62%)
Current	412 (12%)	5371 (15%)
Past	1607 (43%)	10669 (23%)
Binge drink		
No	3297 (92%)	32088 (82%)
Yes	243 (8%)	5615 (18%)
Physical activity		
No	2670 (76%)	25514 (68%)
Yes	870 (24%)	12189 (32%)
Obesity		
No	2636 (74%)	30143 (79%)
Yes	904 (26%)	7560 (21%)
Demographic and other factors		
Gender		
Male	1647 (52%)	15128 (49%)
Female	1893 (48%)	22575 (51%)
Age group		
18–44 years	271 (13%)	15349 (57%)
45–64 years	1128 (34%)	14966 (31%)
65 years or older	2141 (53%)	7388 (12%)
Employment		
No	2703 (73%)	16272 (38%)
Yes	837 (27%)	21431 (62%)
Race		
White	2915 (68%)	26064 (53%)
Latino	268 (15%)	6101 (28%)
Asian	211 (11%)	3730 (13%)
African American	146 (6%)	1808 (6%)
Poverty level		
300% FPL or above	1947 (51%)	23400 (56%)
0–99% FPL	367 (13%)	3654 (13%)
100–199% FPL	689 (21%)	5899 (18%)
200–299% FPL	537 (15%)	4750 (13%)

SPD: serious psychological distress; FPL: federal poverty level.

**Table 2:** Univariate and multiple logistic regression analyses for the relationship between all potential risk factors and heart disease.

Variables	Crude OR	95% CI	*P* value	Adjusted OR	95% CI	*P* value
SPD and behavioral factors						
SPD						
No	1			1		
Yes	2.24	1.81–2.77	<0.0001	2.15	1.70–2.72	<0.0001
Smoking status						
Never	1			1		
Current	1.14	0.95–1.36	0.162	1.11	0.91–1.35	0.317
Past	2.55	2.28–2.85	<0.0001	1.41	1.25–1.57	<0.0001
Binge drink						
No	1			1		
Yes	0.44	0.36–0.54	<0.0001	0.83	0.67–1.03	0.0911
Physical activity						
No	1			1		
Yes	0.68	0.60–0.77	<0.0001	0.77	0.67–0.87	<0.0001
Obesity						
No	1			1		
Yes	1.31	1.18–1.46	<0.0001	1.34	1.19–1.52	<0.0001
Demographic and other factors						
Gender						
Male	1			1		
Female	0.90	0.81–0.99	0.027	0.70	0.63–0.78	<0.0001
Age group						
18–44 years	1			1		
45–64 years	4.80	3.99–5.79	<0.0001	4.0	3.32–4.83	<0.0001
65 years or older	18.66	15.66–22.24	<0.0001	10.98	9.18–13.15	<0.0001
Employment						
No	1			1		
Yes	0.23	0.21–0.26	<0.0001	0.51	0.45–0.59	<0.0001
Race						
White	1			1		
Latino	0.41	0.34–0.49	<0.0001	0.70	0.57–0.86	0.0008
Asian	0.65	0.53–0.81	<0.0001	0.85	0.68–1.07	0.172
African American	0.75	0.60–0.93	0.009	0.88	0.69–1.11	0.263
Poverty level						
300% FPL or above	1			1		
0–99% FPL	1.14	0.97–1.35	0.123	1.25	1.04–1.50	0.018
100–199% FPL	1.21	1.04–1.41	0.013	1.12	0.95–1.31	0.193
200–299% FPL	1.24	1.06–1.44	0.0057	1.04	0.88–1.22	0.657

SPD: serious psychological distress; OR: odds ratio; CI: confidence interval; FPL: federal poverty level.

**Table 3:** Age differences in risk factors for heart disease using multiple logistic regression analyses.

Variable	OR (Age 1)^a^	95% CI	*P* value	OR (Age 2)^b^	95% CI	*P* value	OR (Age 3)^c^	95% CI	*P* value
SPD and behavioral factors									
SPD									
No	1			1			1		
Yes	2.29	1.26–4.17	<0.0001	2.24	1.62–3.09	<0.0001	1.81	1.14–2.88	0.0117
Smoking status									
Never	1			1			1		
Current	1.53	1.0–2.35	0.051	1.07	0.83–1.37	0.608	0.90	0.67–1.21	0.486
Past	1.14	0.71–1.83	0.583	1.44	1.19–1.76	0.0002	1.37	1.18–1.59	<0.0001
Binge drink									
No	1			1			1		
Yes	0.96	0.60–1.53	0.852	0.82	0.61–1.1	0.189	0.73	0.48–1.11	0.138
Physical activity									
No	1			1			1		
Yes	1.00	0.72–1.38	0.993	0.79	0.64–0.98	0.0339	0.67	0.57–0.80	<0.0001
Obesity									
No	1			1			1		
Yes	1.28	0.86–1.92	0.228	1.45	1.17–1.79	0.0006	1.27	1.06–1.52	0.0091
Demographic and other factors									
Gender									
Male	1			1			1		
Female	1.11	0.81–1.54	0.52	0.69	0.57–0.85	0.0003	0.60	0.52–0.70	<0.0001
Employment									
No	1			1			1		
Yes	0.73	0.51–1.06	0.097	0.47	0.39–0.57	<0.0001	0.48	0.36–0.63	<0.0001
Race									
White	1			1			1		
Latino	0.86	0.55–1.32	0.482	0.79	0.58–1.07	0.131	0.54	0.36–0.79	0.0016
Asian	0.99	0.63–1.58	0.994	0.90	0.61–1.32	0.586	0.80	0.56–1.13	0.206
African American	1.34	0.69–2.59	0.383	0.92	0.65–1.30	0.645	0.68	0.49–0.94	0.0191
Poverty level									
300% FPL or above	1			1			1		
0–99% FPL	0.98	0.57–1.71	0.954	1.14	0.81–1.61	0.451	1.58	1.20–2.07	0.001
100–199% FPL	1.41	0.86–2.30	0.17	1.07	0.80–1.42	0.646	1.06	0.88–1.28	0.545
200–299% FPL	0.49	0.25–0.96	0.0387	1.02	0.74–1.40	0.915	1.20	0.98–1.46	0.0713

SPD: serious psychological distress; OR: odds ratio; CI: confidence interval; FPL: federal poverty level.

^a^Age 1: 18–44 years.

^b^Age 2: 45–64 years.

^c^Age 3: 65 years or older.
